# Exploring Serum Vitamin D, Sex Hormones, and Lipid Profile Disparities in Women With and Without Polycystic Ovarian Syndrome: A Case-Control Study

**DOI:** 10.7759/cureus.60975

**Published:** 2024-05-24

**Authors:** Lateef A Akinola, Gideon N Inyangudo, Abimbola T Ottun, Ayokunle M Olumodeji, Adeniyi A Adewunmi, Folasade H Olalere, Olufemi M Omololu, Babalola D Ogungbemile

**Affiliations:** 1 Department of Obstetrics and Gynaecology, Medison Specialist Women's Hospital, Lagos, NGA; 2 Department of Research, Medison Specialist Women's Hospital, Lagos, NGA; 3 Department of Obstetrics and Gynaecology, Lagos State University Teaching Hospital, Lagos, NGA; 4 Department of Obstetrics and Gynaecology, Lagos State University College of Medicine, Lagos, NGA; 5 Department of Obstetrics and Gynaecology, Lagos Island Maternity Hospital, Lagos, NGA

**Keywords:** polycystic ovarian syndrome (pcos), hormonal imbalance, lipid profile, progesterone, testosterone, amh, vitamin d

## Abstract

Background

Polycystic ovarian syndrome (PCOS) is a common endocrine disorder affecting women of reproductive age. It is characterized by dyslipidemia, hormonal imbalances, and metabolic dysfunctions. Vitamin D deficiency may be implicated in the pathogenesis of PCOS, potentially exacerbating its metabolic syndrome. However, the exact interplay between these factors remains underexplored.

Aim

This study aimed to evaluate serum levels of vitamin D and its association with modalities of PCOS among women with PCOS and healthy controls.

Methods

This was a hospital-based case-control study where 60 women newly diagnosed with PCOS and 56 non-PCOS controls were consecutively recruited within a 10-month period. The women aged 20-40 were recruited at the gynecology clinics of Lagos State University Teaching Hospital and Lagos Island Maternity Hospital. PCOS was diagnosed using the Rotterdam’s criteria. The biodata, anthropometry, clinical features, serum vitamin D, cortisol, progesterone, testosterone, estradiol, prolactin, anti-Mullerian hormone (AMH), thyroid-stimulating hormone, follicle-stimulating hormone (FSH), luteinizing hormone (LH), insulin, fasting blood glucose (FBG), total cholesterol (TC), high-density lipoprotein cholesterol (HDL-C), low-density lipoprotein cholesterol (LDL-C), triglyceride (TG), and very-low-density lipoprotein cholesterol (VLDL-C) levels of PCOS-diagnosed women were assessed and compared with those of women without PCOS. The exclusion criteria comprised known diabetics, women with gynecological pathologies such as fibroids, and women on medications affecting the study analytes or hormones. Statistical analyses included chi-square or Fisher’s exact tests for categorical variables, student t-test for continuous variables, and Pearson’s correlation for assessing relationships between continuous variables. The significance level was set at p<0.05 and a confidence interval of 95%.

Results

Individuals with PCOS exhibited a younger mean age (26.90±3.73 versus 29.95±5.00 years, p=0.001) and a higher prevalence of irregular menstrual patterns (46.7% versus 14.3%, p=0.0001) and acne (58.3% versus 37.5%, p=0.025). Moreover, PCOS was associated with elevated levels of TC (p = 0.03), TG (p = 0.03), LDL-C (p = 0.014), FBG (p = 0.001), LH:FSH ratio (p = 0.002), AMH (p = 0.0001), and testosterone (p = 0.003), but low progesterone (p = 0.001) and vitamin D (p = 0.033), alongside a higher incidence of vitamin D deficiency (33.3% versus 26.1%) and insufficiency (66.7% versus 56.5%). Additionally, significant but weak correlations were observed between serum vitamin D levels and waist-hip ratio (r = 0.4, p = 0.016) and FBG (r = -0.4, p = 0.036) in the PCOS group, suggesting potential metabolic implications.

Conclusion

The PCOS subjects in this study had decreased vitamin D and progesterone levels, with elevated concentrations of testosterone, AMH, lipid profile (TC, LDL, and TG), FBG, and LH:FSH ratio. Studies on the therapeutic effect of vitamin D administration in managing PCOS will need to be further evaluated.

## Introduction

Polycystic ovarian syndrome (PCOS) is a common endocrinopathy often diagnosed in women of reproductive age globally [1]. First described by Stein and Leventhal in 1935, its prevalence varies from 5% to 15% based on the diagnostic criteria used [1]. It is a prevalent hormonal disorder with implications for metabolism, reproduction, and mental health [2]. PCOS has been linked to several morbidities, such as obesity, metabolic syndrome, impaired glucose tolerance, type 2 diabetes mellitus, depression, obstructive sleep apnea, endometrial cancer, and non-alcoholic fatty liver disease [1]. Considering the complexity of the condition and its significant impact on quality of life, it is essential to promptly diagnose, screen for potential complications, and implement appropriate management approaches [2].

Although the precise causes of PCOS remain unclear, interactions among predisposing risk factors such as abnormal environmental, metabolic, lifestyle, neuroendocrine, and genetic factors contribute to its etiology [3]. The major symptoms essential for the diagnosis of PCOS are anovulation, polycystic ovary, and clinical or biochemical signs of hyperandrogenism such as acne, hirsutism, and alopecia [4]. Other major presentations of PCOS include infertility, obesity, dyslipidemia, luteinizing hormone (LH) hypersecretion, and metabolic syndromes [5]. 

Vitamin D is primarily synthesized in the skin, with a small percentage (about 10%) obtained through dietary sources [6]. Although the primary function of vitamin D is linked to the metabolism of calcium phosphate, its receptors are present in reproductive organs, including the ovaries, endometrium, and placenta, suggesting that vitamin D may have effects on reproduction [7]. The symptoms of PCOS, including irregular menstruation, decreased chances of getting pregnant, hirsutism, hyperandrogenism, obesity, and elevated risk factors for cardiovascular disease, have been linked to lower vitamin D levels [8]. Nevertheless, inconsistent reports on the prevalence of vitamin D deficiency in PCOS patients have been documented [9-11].

 Studies reporting vitamin D deficiency among PCOS women have been on the increase. However, there is a paucity of studies assessing vitamin D levels among PCOS women in sub-Saharan Africa. Hence, this study assessed serum vitamin D levels in addition to sex hormones and lipid profiles among women diagnosed with PCOS in Lagos state, Nigeria. This study population which comprises women in the sub-Saharan African region addresses a gap in the existing literature.

## Materials and methods

Study design

This study compared the differences in serum vitamin D, sex hormones, and lipid profiles between women with and without PCOS, employing a prospective case-control study design. The study was conducted at the gynecology clinics of Lagos Island Maternity Hospital and Lagos State University Teaching Hospital over a 10-month period.

Study population

The study population comprised 60 women newly diagnosed with PCOS and 56 non-PCOS controls, consecutively recruited during the study period. Written informed consent was obtained from all the participants before data collection. The 2003 Rotterdam criteria, which require the presence of at least two of the following characteristics: anovulation or oligo-ovulation, clinical and/or biochemical hyperandrogenism, and polycystic ovarian morphology on ultrasound, were used to make the PCOS diagnosis [4]. Non-PCOS controls were selected based on the absence of any criteria indicative of PCOS, including regular menstrual cycles.

Inclusion criteria

Newly diagnosed PCOS women aged between 20 and 40 years were recruited as PCOS subjects, while healthy women of similar age ranges were recruited as controls.

Exclusion criteria

Women excluded from this study include nursing mothers, diabetic women, PCOS patients undergoing treatment, and patients receiving therapy for hormone replacement. Additionally, women with hormonal abnormalities, fibroids, and other gynecological pathologies of the uterus and ovaries were excluded.

Data/sample collection

Data collection involved comprehensive assessments of participants' biodata, anthropometry, and clinical features of PCOS. Additionally, 10 ml of venous blood was drawn from each participant to assess concentrations of vitamin D and hormones, including testosterone, progesterone, thyroid-stimulating hormone (TSH), prolactin, anti-Mullerian hormone (AMH), follicle-stimulating hormone (FSH), estradiol, cortisol, insulin, and luteinizing hormone (LH), as well as lipid profiles such as total cholesterol, low-density lipoprotein cholesterol (LDL-C), high-density lipoprotein cholesterol (HDL-C), very low-density lipoprotein (VLDL-C), triglycerides, and fasting blood glucose (FBG). The collected blood samples were put into tubes containing fluoride oxalate and clot activator separation gel. The specimens were transported to the laboratory within three hours of collection. The specimen tubes were centrifuged at 3000 rpm for 15 minutes for serum extraction. Serum for FBG was analyzed, while those for vitamin D, hormonal profile, and lipid profile were kept at -200°C and subsequently used for biochemical assays.

Biochemical analysis

Hormones including LH, FSH, testosterone, prolactin, cortisol, insulin, progesterone, TSH, thyroxine (T4), AMH, estradiol, and Vitamin D were analyzed using BioMérieux Mini Vidas automated multi-parametric immunoassay kits for enzyme-linked immunosorbent assay (BioMérieux 376, Chemin De l’Orme, 69280 Marcy l'Etoile, France). The Erba Mannheim Autoanalyzer (Model XL-180) was used to analyze the following: serum total cholesterol and triglycerides, HDL level, and FBG level (Erba Mannheim Corporate Services, Limited, Gw1 Great Western House Great West Road, tw89df, London, United Kingdom of Great Britain and Northern Ireland). The serum concentrations of VLDL and LDL-C were computed using the Friedewald formula [12]. Vitamin D was classified using the previous classification by Hollick et al. [13].

Data analysis

IBM SPSS Statistics for Windows, Version 21 (Released 2012; IBM Corp., Armonk, New York, United States) was used to conduct statistical analyses. The chi-square or Fisher’s exact tests were used for categorical variables and Student's t-test for continuous variables. Pearson’s correlation was used to assess the relationship between continuous variables. The level of statistical significance was set at a p-value of less than 0.05 at a 95% confidence interval. Additionally, adjustments for potential confounders were made where necessary to ensure the robustness and reliability of the study findings.

Ethical considerations

The study was conducted in accordance with the principles of the Helsinki Declaration. Ethical approval with protocol number LREC/06/10/1853 was obtained from the Health Research and Ethics Committee of Lagos State University Teaching Hospital.

## Results

Table 1 presents a comprehensive comparison of anthropometric and clinical characteristics between the study populations, comprising individuals with and without PCOS. Between the two groups, there was a notable difference in the age distribution (p = 0.001), with the PCOS group exhibiting a younger mean age of 26.90± 3.73 years compared to 29.95±5.00 years in the non-PCOS group. Regarding the body mass index (BMI), the PCOS cohort displayed a slightly higher mean BMI of 26.39±4.34 kg/m² compared to 25.55±5.04 kg/m² in the non-PCOS cohort. However, the observed difference was not significant (p = 0.338). Further analysis revealed no significant disparity in BMI grade distribution (p = 0.178), with both groups exhibiting similar proportions across underweight, normal weight, overweight, and obesity categories (Table 1).

**Table 1:** Comparison of anthropometric and clinical characteristics in the study population *t-test applied, ^¥^Fischer’s exact test applied, ^#^Chi-square applied

Variable	PCOS (n=60)	Non-PCOS (n=56)	p-value
Age (years)	26.90 ± 3.73	29.95 ± 5.00	0.001*
BMI (kg/m^2^)	26.39±4.34	25.55±5.04	0.338*
BMI grade			0.178^¥^
Underweight	2 (3.3%)	1 (1.8%)	
Normal weight	20 (33.3%)	30 (53.6%)	
Overweight	25 (41.7%)	17 (30.4%)	
Obesity	13 (21.7%)	8 (14.3%)	
Weight (kg)	69.06 ± 11.31	66.30 ± 11.56	0.197*
Waist-hip ratio	0.83± 0.09	0.84± 0.05	0.525*
Menstrual pattern			0.0001^#^
Irregular	28 (46.7%)	8 (14.3%)	
Regular	32 (53.3%)	48 (85.7%)	
Hirsutism			0.168^¥^
Present	2 (3.3%)	0 (0%)	
Absent	58 (96.7%)	56 (100%)	
Alopecia			0.944^¥^
Present	2 (3.3%)	2 (3.6%)	
Absent	58 (96.7%)	54 (96.4%)	
Acne			0.025^#^
Present	35 (58.3%)	21 (37.5%)	
Absent	25 (41.7%)	35 (62.5%)	

Furthermore, this study assessed various clinical parameters, including menstrual patterns, hirsutism, alopecia, and acne (Table 1). Results indicated a significant association between PCOS and irregular menstrual patterns (p = 0.0001), with 46.7% of PCOS individuals experiencing irregular cycles compared to only 14.3% in the non-PCOS group. Additionally, the presence of acne in the PCOS cohort was observed to be significantly higher (58.3%) compared to the cohort without PCOS (37.5%) (p = 0.025), underscoring the dermatological manifestations associated with PCOS. However, between the two groups, there were no statistically significant variations in hirsutism, alopecia, or the waist-hip ratio (WHR) (Table 1).

A detailed comparison of serum vitamin D, glucose, thyroid, lipid, and reproductive hormone profiles between individuals with PCOS and those without PCOS is depicted in Table 2. Significant differences were observed in several parameters, including total cholesterol (TC), triglyceride (TG), HDL-C, LDL-C, FBG, vitamin D level, testosterone, progesterone, LH:FSH ratio, AMH, and insulin levels. Notably, individuals with PCOS exhibited lower means of vitamin D (20.315 ± 4.81 ng/ml vs. 24.478 ± 8.03 ng/ml, p = 0.033) and progesterone (2.421± 4.64 ng/ml vs. 5.210± 6.82 ng/ml, p = 0.001) compared to the non-PCOS group. Furthermore, participants in the PCOS group exhibited higher mean levels of TC (5.008 ± 1.00 mmol/L vs. 4.435± 0.82 mmol/L, p = 0.003), LDL-C (3.102 ± 0.89 mmol/L vs. 2.667 ± 0.82 mmol/L, p = 0.014), TG (1.274 mmol/L ± 0.60 vs. 0.947 mmol/L ± 0.43, p = 0.003), FBG (6.064 ± 1.98 mmol/L vs. 4.924 ± 1.78 mmol/L, p = 0.001), and testosterone (0.383± 0.29 mol/L vs. 0.213± 0.18 mol/L, p = 0.003) compared to non-PCOS individuals. Additionally, PCOS individuals exhibited a higher prevalence of vitamin D deficiency (33.3% vs. 26.1%) and insufficiency (66.7% vs. 56.5%) compared to non-PCOS individuals (Table 2).

**Table 2:** Comparison of serum vitamin D, glucose, thyroid, lipid and reproductive hormone profiles in the study population *t-test applied, ^¥^Fischer’s exact test applied; ^#^Chi-square applied AMH: Anti-Mullerian hormone; TSH: thyroid-stimulating hormone, FSH: follicle-stimulating hormone, LH: luteinizing hormone, Insulin, FBG: fasting blood glucose, TC: total cholesterol, HDL-C: high-density lipoprotein cholesterol, LDL-C: low-density lipoprotein cholesterol, TG: triglyceride, VLDL-C: very low-density lipoprotein cholesterol; PCOS: polycystic ovarian syndrome

Variables	PCOS (n=60) Mean ± SD	Non-PCOS (n=56) Mean ± SD	p-value
Total cholesterol (mmol/L)	5.008 ± 1.00	4.435 ± 0.82	0.003*
Triglyceride (mmol/L)	1.274 ± 0.60	0.947 ± 0.43	0.003*
HDL-C (mmol/L)	1.359 ± 0.48	1.353 ± 0.46	0.0001*
LDL-C (mmol/L)	3.102 ± 0.89	2.667 ± 0.82	0.014*
LDL:HDL (mmol/L)	2.552 ± 1.26	2.484 ± 1.67	0.819*
VLDL-C (mmol/L)	0.258 ± 0.12	0.227 ± 0.26	0.458*
FBG (mmol/L)	6.064 ± 1.98	4.924 ± 1.78	0.001*
Vitamin D level (ng/ml)	20.315 ± 4.81	24.478 ± 8.03	0.033*
Vitamin D grades			0.05^¥^
Deficiency	33.3%	26.1%	
Insufficiency	66.7%	56.5%	
Sufficient	0%	17.4%	
Testosterone (mol/L)	0.383 ± 0.29	0.213 ± 0.18	0.003*
TSH (ng/ml)	1.850 ± 1.00	2.193 ± 0.83	0.109*
T4 (pmol/L)	15.315 ±2.28	14.852 ±3.01	0.434*
Prolactin (ng/ml)	17.910 ± 11.15	19.501 ± 22.08	0.636*
Progesterone (ng/ml)	2.421 ± 4.64	5.210 ± 6.82	0.001*
LH (mUI/ml)	9.907 ± 11.88	7.234 ± 8.19	0.203*
FSH (mUI/ml)	5.127 ± 3.48	9.730 ± 16.83	0.071*
LH:FSH	2.201 ± 2.22	1.014 ± 0.81	0.002*
AMH (ng/ml)	5.990 ± 2.49	2.420 ± 2.30	0.0001*
Estradiol II (pmol/L)	88.315 ± 60.77	101.161 ± 100	0.597*
Insulin (miu/L)	30.437 ± 27.76	21.294 ± 16.03	0.229*
Cortisol	5.187 ± 1.93	5.280 ± 3.30	0.889*

Moreover, the study revealed no significant differences in VLDL-C, LDL:HDL ratio, TSH, T4, prolactin, LH, FSH, estradiol II, insulin, or cortisol levels between the two groups. Nonetheless, the PCOS group exhibited significantly higher levels of AMH (5.990 ± 2.49 ng/ml vs. 2.420 ± 2.30 ng/ml, p = 0.0001) and the LH:FSH ratio (2.201 ± 2.22 vs. 1.014 ± 0.81, p = 0.002), highlighting the hormonal imbalances associated with the syndrome (Table 2).

 Correlations between serum vitamin D levels and various clinical and biochemical parameters in both PCOS and non-PCOS women are presented in Table 3. In the PCOS group, significant correlations were found between vitamin D levels and WHR (r = 0.415, p = 0.016) as well as FBG (r = -0.367, p = 0.036), indicating a positive association with WHR and a negative association with FBG (Table 3). Notably, cortisol and serum vitamin D levels showed a significant positive correlation in the non-PCOS group (r = 0.493, p = 0.032), suggesting a potential hormonal influence on vitamin D metabolism. Conversely, no significant correlations were noted between vitamin D levels and age, BMI, reproductive hormones (progesterone, testosterone, estradiol, prolactin, AMH, TSH, FSH, LH, LH/FSH ratio), lipid profile (TC, HDL-C, LDL-C, TG, VLDL-C, LDL-HDL ratio), or insulin levels in either group (Table 3).

**Table 3:** Correlation of serum vitamin D levels with clinical and biochemical parameters in PCOS and non-PCOS women r: Pearson correlation; AMH: Anti-Mullerian hormone, TSH: thyroid-stimulating hormone, FSH: follicle stimulating hormone, LH: luteinizing hormone, Insulin, FBG: fasting blood glucose, TC: total cholesterol, HDL-C: high-density lipoprotein cholesterol, LDL-C: low-density lipoprotein cholesterol, TG: triglyceride; VLDL-C: very low-density lipoprotein; PCOS: polycystic ovarian syndrome

Variables	PCOS (n=60)		Non-PCOS (n=56)	
	r	p-value	r	p-value
Age (years)	-0.141	0.435	-0.122	0.581
BMI (Kg/M^2^)	-0.210	0.241	-0.123	0.577
Waist-hip ratio	0.415	0.016	0.058	0.793
Cortisol	-0.392	0.108	0.493	0.032
Progesterone (ng/ml)	0.381	0.07	0.182	0.442
Testosterone (mol/l)	0.091	0.691	0.391	0.072
Estradiol (pmol/l)	0.136	0.591	0.509	0.133
Prolactin (ng/ml)	-0.025	0.894	0.201	0.370
AMH (ng/ml)	0.165	0.366	-0.370	0.082
TSH (ng/ml)	-0.065	0.758	0.412	0.090
FSH (mUI/ml)	-0.008	0.966	-0.256	0.238
LH (mUI/ml)	-0.030	0.870	-0.143	0.524
LH/FSH	-0.041	0.820	-0.256	0.238
Insulin (mu/L)	-0.203	0.390	0.594	0.214
FBG (mmol/dl)	-0.367	0.036	0.041	0.853
TC (mmol/L)	-0.147	0.429	-0.034	0.887
HDL-C (mmol/L)	-0.105	0.574	-0.221	0.349
LDL-C (mmol/L)	-0.075	0.687	0.276	0.239
TG (mmol/L)	-0.123	0.510	-0.062	0.794
VLDL-C (mmol/L)	-0.106	0.557	0.066	0.775
LDL-HDL	0.009	0.962	0.133	0.577

## Discussion

The study compared anthropometric, clinical, and biochemical characteristics between individuals with and without PCOS. Our study findings align with prior published works on PCOS but also diverge from previous research regarding several key aspects of PCOS.

Unlike Sukul et al. and Ezeiruka and Onitsha, who reported a significant disparity in BMI between PCOS subjects and controls [9,14], our study, aligning with Ansari and Chandra [10] and Bindayel [15], observed no significant BMI difference between the two groups. Lifestyle disparities among study populations may contribute to this inconsistency. Notably, acne prevalence was significantly higher in PCOS-affected women in our study compared to controls; this observation was consistent with the findings of Ansari and Chandra [10] and Yadav and Tarware [16]. Similarly, the increased incidence of irregular menstrual cycles in the PCOS group is in line with the clinical features typical of PCOS [2] and agrees with a previous report by Yadav and Tarware [16].

Furthermore, our study revealed significantly higher levels of TC, TG, and LDL-C in PCOS subjects compared to controls, consistent with previous research indicating dyslipidemia as a potential complication of PCOS [9,17,18]. The higher mean blood glucose in the PCOS group observed in this study reflects their elevated risk of hyperinsulinemia and diabetes mellitus [19], contrasting with the findings of Bindayel and Kim et al. [15,20] but agreeing with the findings of Kharb et al. [19] and Eftekhar et al. [21]. This supports the suggestion that hyperinsulinemia may be linked to PCOS pathophysiology.

Vitamin D deficiency has been suggested to contribute to the pathogenesis of insulin resistance and PCOS [10]. Vitamin D deficiency, implicated in PCOS pathogenesis, was evident in our study, aligning with prior research [9,15,22]. However, discordant findings regarding vitamin D levels among PCOS subjects exist in the literature [10,11,20], underscoring the complexity of this relationship.

In agreement with the findings of Eftekhar et al. and Oladosu et al., the elevated AMH serum concentration was also observed in the PCOS group in our study [21,23]. The combination of AMH levels and other clinical symptoms has been suggested to be an effective criterion for diagnosing PCOS. In known PCOS patients, the combination of hyperandrogenism and AMH levels was found to have a high sensitivity and specificity for PCOS diagnosis [24]. The ovaries of PCOS patients are characterized by an increased antral follicle count, which may cause increased AMH production per follicle [23,24]. Serum testosterone concentrations in this study were significantly higher in PCOS patients, consistent with hyperandrogenemia, a hallmark of PCOS [1,2]. This observed hyperandrogenism was in accordance with earlier studies by Ezeiruaka and Onitsha [14] and Oladoso et al. [23]. Conversely, serum progesterone levels consistent with previous studies [14,25] were significantly lower in the PCOS group, indicating potential ovulatory dysfunction.

In line with the report by Lv et al. [26], estradiol levels in our study did not significantly differ between groups. However, this result disagreed with earlier findings by Ezeiruaka and Onitsha and Oyebanji et al. [14,25]. The age range of the subjects in this study may account for this observed inconsistency. While LH and FSH levels in this study did not significantly differ between groups, the LH/FSH ratio was notably higher in PCOS subjects, indicative of hormonal imbalance and similar to some previous reports [14,26,27]. Prolactin levels showed no significant difference between groups. 

Finally, significant but weak correlations between vitamin D levels and WHR were found in the PCOS group (WHR) (r = 0.415, p = 0.016) as well as FBG (r = -0.367, p = 0.036), indicating a positive association with WHR and a negative association with FBG. Notably, there was a weak but statistically significant positive correlation between cortisol and serum vitamin D levels in the non-PCOS group (r = 0.493, p = 0.032), suggesting a potential hormonal influence on vitamin D metabolism. Conversely, no significant correlations were noted between vitamin D levels and age, BMI, reproductive hormones (progesterone, testosterone, estradiol, prolactin, AMH, TSH, FSH, LH, LH/FSH ratio), lipid profile (TC, HDL-C, LDL-C, TG, VLDL-C, LDL-HDL ratio), or insulin levels in either group.

Strengths of the study

Testing all the hormones and analytes in the same study population is a strength because it allows for a comprehensive understanding of the interplay between these factors within the same context, providing a more holistic view of PCOS pathophysiology. This approach avoids potential confounding variables related to different study populations and enhances the ability to derive more precise and nuanced deductions about the relationships between these factors in PCOS.

Limitations

The limited sample size in this study may have led to insufficient statistical power to identify certain significant relationships as statistically significant. Therefore, more research with a larger sample size is required to validate and confirm our findings.

## Conclusions

Our results suggest that serum vitamin D levels are significantly lower in women diagnosed with PCOS than in women without PCOS. Our study also showed that serum vitamin D concentration in the PCOS group is correlated with the WHR and FBG. Additionally, we observed a weak and non-significant correlation between vitamin D and other biochemical, metabolic, and clinical parameters. The research on the therapeutic effect of vitamin administration in PCOS management will need to be further evaluated.
